# Supplementary material for: Flagellar energetics from high-resolution imaging of beating patterns in tethered mouse sperm
Source: eLife. 2021 Apr 30;10:e62524. doi: 10.7554/eLife.62524 (PMC8159377; doi:10.7554/eLife.62524)
Supplement: Transparent reporting form [file elife-62524-transrepform.docx]

***eLife’s* transparent reporting form**

We encourage authors to provide detailed information *within their submission* to facilitate the interpretation and replication of experiments. Authors can upload supporting documentation to indicate the use of appropriate reporting guidelines for health-related research (see [EQUATOR Network](about:blank)), life science research (see the [BioSharing Information Resource](about:blank)), or the [ARRIVE guidelines](about:blank) for reporting work involving animal research. Where applicable, authors should refer to any relevant reporting standards documents in this form.

If you have any questions, please consult our Journal Policies and/or contact us: [editorial@elifesciences.org](about:blank).

**Sample-size estimation**

- You should state whether an appropriate sample size was computed when the study was being designed
- You should state the statistical method of sample size computation and any required assumptions
- If no explicit power analysis was used, you should describe how you decided what sample (replicate) size (number) to use

Please outline where this information can be found within the submission (e.g., sections or figure legends), or explain why this information doesn’t apply to your submission:

In this study, we use two independent approaches to defining the sample size. The first approach was to define each movie as a sample and perform the statistical analysis. In this case N = 5 (WT) and N = 5 (*Crisp2* KO). Since this number is small, the statistical tests comparing the two populations were not conclusive. Further a one-way ANOVA revealed large sample-to-sample variation within each population leading to large standard deviations. The second approach was to treat each individual time-cycle as a sample and pool all the time-cycles in the population together, thus creating a much larger sample set. This large sample set (N~300) allows for conclusive statistical inferences to be drawn.

The same is mentioned in line numbers 342 to 370 in the manuscript.

**Replicates**

- You should report how often each experiment was performed
- You should include a definition of biological versus technical replication
- The data obtained should be provided and sufficient information should be provided to indicate the number of independent biological and/or technical replicates
- If you encountered any outliers, you should describe how these were handled
- Criteria for exclusion/inclusion of data should be clearly stated
- High-throughput sequence data should be uploaded before submission, with a private link for reviewers provided (these are available from both GEO and ArrayExpress)

Please outline where this information can be found within the submission (e.g., sections or figure legends), or explain why this information doesn’t apply to your submission:

The beating of each sperm cell was recorded for 10s to produce one sample movie. 40-60 time cycles were recorded for each cell. Cells from 3 animals were used in the WT and 5 animals in the *Crisp2* KO populations. The data exclusion criteria are described in lines 477 to 483. Movies with more than 10% deviation from the average contour length were discarded. These were movies with imaging/image processing errors. Also, two *Crisp2* KO movies with outlier mean tangent angle profiles were not discussed since they did not change the main conclusions of the paper regarding the flagellar energetics.

The same is described in line numbers 342 to 370 in the manuscript.

**Statistical reporting**

- Statistical analysis methods should be described and justified
- Raw data should be presented in figures whenever informative to do so (typically when N per group is less than 10)
- For each experiment, you should identify the statistical tests used, exact values of N, definitions of center, methods of multiple test correction, and dispersion and precision measures (e.g., mean, median, SD, SEM, confidence intervals; and, for the major substantive results, a measure of effect size (e.g., Pearson's r, Cohen's d)
- Report exact p-values wherever possible alongside the summary statistics and 95% confidence intervals. These should be reported for all key questions and not only when the p-value is less than 0.05.

Please outline where this information can be found within the submission (e.g., sections or figure legends), or explain why this information doesn’t apply to your submission:

Figure 4C and Figure 5D show the distribution of the raw data. The statistical tests used are described in the figure captions. The results of the statistical tests and p-values are reported in Table 1, Table 2, and Table 3 in the Supplementary Information. The one-way ANOVA and the unpaired two-tailed Student t-test were performed in Matlab.

(For large datasets, or papers with a very large number of statistical tests, you may upload a single table file with tests, Ns, etc., with reference to sections in the manuscript.)

**Group allocation**

- Indicate how samples were allocated into experimental groups (in the case of clinical studies, please specify allocation to treatment method); if randomization was used, please also state if restricted randomization was applied
- Indicate if masking was used during group allocation, data collection and/or data analysis

Please outline where this information can be found within the submission (e.g., sections or figure legends), or explain why this information doesn’t apply to your submission:

In this study the two experimental group were studied - Wild type (WT) which are normal adult male mice and *Crisp2* knock out (KO) which are adult male mice in which the *Crisp2* gene was genetically knock-out.

**Additional data files (“source data”)**

- We encourage you to upload relevant additional data files, such as numerical data that are represented as a graph in a figure, or as a summary table
- Where provided, these should be in the most useful format, and they can be uploaded as “Source data” files linked to a main figure or table
- Include model definition files including the full list of parameters used
- Include code used for data analysis (e.g., R, MatLab)
- Avoid stating that data files are “available upon request”

Please indicate the figures or tables for which source data files have been provided:

The raw dataset available at the DOI: [10.26180/5f50562bb322b](about:blank) . Matlab code is currently being streamlined and cleaned up and can be made available if necessary. The key algorithms are described in the manuscript, in the section ‘Materials and Methods’.
